# Asymptomatic versus Symptomatic Patients with Severe Aortic Stenosis

**DOI:** 10.1038/s41598-018-28162-x

**Published:** 2018-07-04

**Authors:** Norio Kanamori, Tomohiko Taniguchi, Takeshi Morimoto, Hiroki Shiomi, Kenji Ando, Koichiro Murata, Takeshi Kitai, Yuichi Kawase, Chisato Izumi, Makoto Miyake, Hirokazu Mitsuoka, Masashi Kato, Yutaka Hirano, Shintaro Matsuda, Kazuya Nagao, Tsukasa Inada, Hiroshi Mabuchi, Yasuyo Takeuchi, Keiichiro Yamane, Mamoru Toyofuku, Mitsuru Ishii, Eri Minamino-Muta, Takao Kato, Moriaki Inoko, Tomoyuki Ikeda, Akihiro Komasa, Katsuhisa Ishii, Kozo Hotta, Nobuya Higashitani, Yoshihiro Kato, Yasutaka Inuzuka, Chiyo Maeda, Toshikazu Jinnai, Yuko Morikami, Naritatsu Saito, Kenji Minatoya, Takeshi Aoyama, Takeshi Kimura, CURRENT AS registry Investigators, Masao Imai, Junichi Tazaki, Toshiaki Toyota, Hirooki Higami, Tetsuma Kawaji, Shinichi Shirai, Kengo Kourai, Takeshi Arita, Shiro Miura, Kyohei Yamaji, Tomoya Onodera, Yutaka Furukawa, Kitae Kim, Kazushige Kadota, Keiichiro Iwasaki, Hiroshi Miyawaki, Ayumi Misao, Akimune Kuwayama, Masanobu Ohya, Takenobu Shimada, Hidewo Amano, Yoshihisa Nakagawa, Masashi Amano, Yusuke Takahashi, Yusuke Yoshikawa, Shunsuke Nishimura, Maiko Kuroda, Manabu Shirotani, Shinji Miki, Tetsu Mizoguchi, Takafumi Yokomatsu, Akihiro Kushiyama, Hidenori Yaku, Toshimitsu Watanabe, Shunichi Miyazaki, Naoki Takahashi, Kohei Fukuchi, Tomoyuki Murakami, Teruki Takeda, Tomoko Sakaguchi, Keiko Maeda, Masayuki Yamaji, Motoyoshi Maenaka, Yutaka Tadano, Hiroki Sakamoto, Makoto Motooka, Ryusuke Nishikawa, Hiroshi Eizawa, Mitsunori Kawato, Minako Kinoshita, Kenji Aida, Takashi Tamura, Kousuke Takahashi, Euihong Ko, Masaharu Akao, Nobutoyo Masunaga, Hisashi Ogawa, Moritake Iguchi, Takashi Unoki, Kensuke Takabayashi, Yasuhiro Hamatani, Yugo Yamashita, Yoshihiro Himura, Yukihito Sato, Shuhei Tsuji, Takashi Konishi, Kouji Sogabe, Michiya Tachiiri, Yukiko Matsumura, Chihiro Ota, Ichiro Kouchi, Shigeru Ikeguchi, Soji Nishio, Jyunya Seki, Eiji Shinoda, Miho Yamada, Akira Kawamoto, Shoji Kitaguchi, Ryuzo Sakata, Mitsuo Matsuda, Sachiko Sugioka, Yuji Hiraoka, Michiya Hanyu, Fumio Yamazaki, Tadaaki Koyama, Tatsuhiko Komiya, Kazuo Yamanaka, Noboru Nishiwaki, Hiroyuki Nakajima, Motoaki Ohnaka, Hiroaki Osada, Katsuaki Meshii, Toshihiko Saga, Masahiko Onoe, Shogo Nakayama, Genichi Sakaguchi, Atsushi Iwakura, Kotaro Shiraga, Koji Ueyama, Keiichi Fujiwara, Atsushi Fukumoto, Senri Miwa, Junichiro Nishizawa, Mitsuru Kitano

**Affiliations:** 1 Division of Cardiology, Shimada Municipal Hospital, Shimada, Japan; 2 Department of Cardiovascular Medicine, Kyoto University Graduate School of Medicine, Kyoto, Japan; 3 Department of Clinical Epidemiology, Hyogo College of Medicine, Nishinomiya, Japan; 4 Department of Cardiology, Kokura Memorial Hospital, Kokura, Japan; 5 Department of Cardiology, Shizuoka City Shizuoka Hospital, Shizuoka, Japan; 6 Department of Cardiovascular Medicine, Kobe City Medical Center General Hospital, Kobe, Japan; 7 Department of Cardiovascular Medicine, Kurashiki Central Hospital, Kurashiki, Japan; 8 Department of Cardiology, Tenri Hospital, Tenri, Japan; 9 Division of Cardiology, Nara Hospital, Kinki University Faculty of Medicine, Ikoma, Japan; 10 Department of Cardiology, Mitsubishi Kyoto Hospital, Kyoto, Japan; 11 Department of Cardiology, Kinki University Hospital, Osakasayama, Japan; 12 Department of Cardiovascular Center, Osaka Red Cross Hospital, Osaka, Japan; 13 Department of Cardiology, Koto Memorial Hospital, Higashiomi, Japan; 14 Department of Cardiology, Shizuoka General Hospital, Shizuoka, Japan; 15 Department of Cardiology, Nishikobe Medical Center, Kobe, Japan; 16 Department of Cardiology, Japanese Red Cross Wakayama Medical Center, Wakayama, Japan; 17 Department of Cardiology, National Hospital Organization Kyoto Medical Center, Kyoto, Japan; 18 Cardiovascular Center, The Tazuke Kofukai Medical Research Institute, Kitano Hospital, Osaka, Japan; 19 Department of Cardiology, Hikone Municipal Hospital, Hikone, Japan; 20 Department of Cardiology, Kansai Electric Power Hospital, Osaka, Japan; 21 Department of Cardiology, Hyogo Prefectural Amagasaki General Medical Center, Amagasaki, Japan; 22 Department of Cardiology, Japanese Red Cross Otsu Hospital, Otsu, Japan; 23 Department of Cardiology, Saiseikai Noe Hospital, Osaka, Japan; 24 Department of Cardiology, Shiga Medical Center for Adults, Moriyama, Japan; 25 Department of Cardiology, Hamamatsu Rosai Hospital, Hamamatsu, Japan; 26 Department of Cardiology, Hirakata Kohsai Hospital, Hirakata, Japan; 27 Department of Cardiovascular Surgery, Kyoto University Graduate School of Medicine, Kyoto, Japan; 28 Department of Cardiology, Kishiwada City Hospital, Kishiwada, Japan; 29 Department of Cardiology, Rakuwakai Otowa Hospital, Kyoto, Japan; 30 Department of Cardiovascular Surgery, Kokura Memorial Hospital, Kitakyushu, Japan; 31 Department of Cardiovascular Surgery, Shizuoka City Shizuoka Hospital, Shizuoka, Japan; 32 Department of Cardiovascular Surgery, Kobe City Medical Center General Hospital, Kobe, Japan; 33 Department of Cardiovascular Surgery, Kurashiki Central Hospital, Kurashiki, Japan; 34 Department of Cardiovascular Surgery, Tenri Hospital, Tenri, Japan; 35 Department of Cardiovascular Surgery, Nara Hospital, Kinki University Faculty of Medicine, Kyoto, Japan; 36 Department of Cardiovascular Surgery, Mitsubishi Kyoto Hospital, Kyoto, Japan; 37 Department of Cardiovascular Surgery, Kinki University Hospital, Osakasayama, Japan; 38 Department of Cardiovascular Surgery, Kishiwada City Hospital, Kishiwada, Japan; 39 Department of Cardiovascular Surgery, Osaka Red Cross Hospital, Osaka, Japan; 40 Department of Cardiovascular Surgery, Shizuoka General Hospital, Shizuoka, Japan; 41 Department of Cardiovascular Surgery, Japanese Red Cross Wakayama Medical Center, Wakayama, Japan; 42 Department of Cardiovascular Surgery, National Hospital Organization Kyoto Medical Center, Kyoto, Japan; 43 Department of Cardiovascular Surgery, Cardiovascular Center, The Tazuke Kofukai Medical Research Institute, Kitano Hospital, Osaka, Japan; 44 Department of Cardiovascular Surgery, Hyogo Prefectural Amagasaki General Medical Center, Amagasaki, Japan; 45 Department of Cardiovascular Surgery, Rakuwakai Otowa Hospital, Kyoto, Japan; 46 Department of Cardiovascular Surgery, Shiga Medical Center for Adults, Moriyama, Japan; 47 Department of Cardiovascular Surgery, Hamamatsu Rosai Hospital, Hamamatsu, Japan; 48 Department of Cardiovascular Surgery, Japanese Red Cross Otsu Hospital, Otsu, Japan

## Abstract

It is unknown how much different are the clinical outcomes between asymptomatic and symptomatic patients with severe aortic stenosis (AS). In the CURRENT AS registry enrolling 3,815 consecutive patients with severe AS, we compared the long-term outcomes between 1808 asymptomatic and 1215 symptomatic patients (exertional dyspnea: N = 813, syncope: N = 136, and angina: N = 266) without heart failure (HF) hospitalization. Symptomatic patients had greater AS severity, and more depressed left ventricular function than asymptomatic patients without much difference in other baseline characteristics. During a median follow-up of 3.2 years, aortic valve replacement (AVR) was performed in 62% of symptomatic patients, and 38% of asymptomatic patients. The cumulative 5-year incidences for the primary outcome measure (a composite of aortic valve-related death or HF hospitalization) was higher in symptomatic patients than in asymptomatic patients (32.3% versus 27.6%, P < 0.001). After adjusting for AVR and other variables, the greater risk of symptomatic relative to asymptomatic patients for the primary outcome measure was significant (hazard ratio 1.64, 95% confidence interval 1.41–1.96, P < 0.001). In conclusions, the excess risk of symptomatic relative to asymptomatic patients with severe AS for the aortic valve-related event was significant. However, the prevalence of AVR in symptomatic patients was not optimal.

## Introduction

Aortic stenosis (AS) is one of the most common valvular heart diseases in the developed countries and its prevalence is increasing in the aging societies^1–3^. Ross and Braunwald reported that the average survival durations of patients with AS after development of symptoms such as angina, syncope, and dyspnea were only 5, 3 and 2 years, respectively^4^. Poor clinical outcomes for conservatively managed patients with symptomatic severe AS have been confirmed^5–9^. Therefore, the current guidelines recommend aortic valve replacement (AVR) for patients with symptomatic severe AS, while the watchful waiting strategy for AVR until symptoms emerge is recommended in patients with asymptomatic severe AS based on the good prognosis of AS patients while they are asymptomatic^10,11^. However, severity of symptoms varies widely in patients with symptomatic severe AS. Prognosis of severe AS patients complicated with acute heart failure (AHF) has been reported to be very poor both in patients undergoing AVR and in patients managed conservatively, while there was no previous study evaluating how much different are the clinical outcomes between asymptomatic and symptomatic patients with severe AS^12^. The comparison between symptomatic and asymptomatic patients with severe AS would be important, because the watchful waiting strategy is actually waiting for the emergence of symptoms to safely provide the opportunity for elective AVR. Therefore, we sought to compare the baseline clinical and echocardiographic characteristics as well as the long-term clinical outcomes between asymptomatic and symptomatic patients excluding those patients with AHF at baseline in a large Japanese observational database of patients with severe AS.

## Methods

### Study population

The study design, methodologies, and outcomes from the CURRENT AS (Contemporary outcomes after sURgery and medical tREatmeNT in patients with severe Aortic Stenosis) registry have been described previously^13–20^. Briefly, the CURRENT AS registry is a retrospective, multicenter registry that enrolled 3815 consecutive patients with severe AS among 27 centers in Japan between January 2003 and December 2011. We searched the hospital database of transthoracic echocardiography, and enrolled consecutive patients who met the definition of severe AS (peak aortic jet velocity [Vmax] > 4.0 m/s, mean aortic pressure gradient [PG] > 40 mm Hg, or aortic valve area [AVA] < 1.0 cm^2^) for the first time during the study period^10,11^. All the Institutional Review Boards approved the protocol. Written informed consent was waived because of the retrospective nature of this study, and no patients refused to participate in the study when contacted for follow-up.

**Figure 1:**
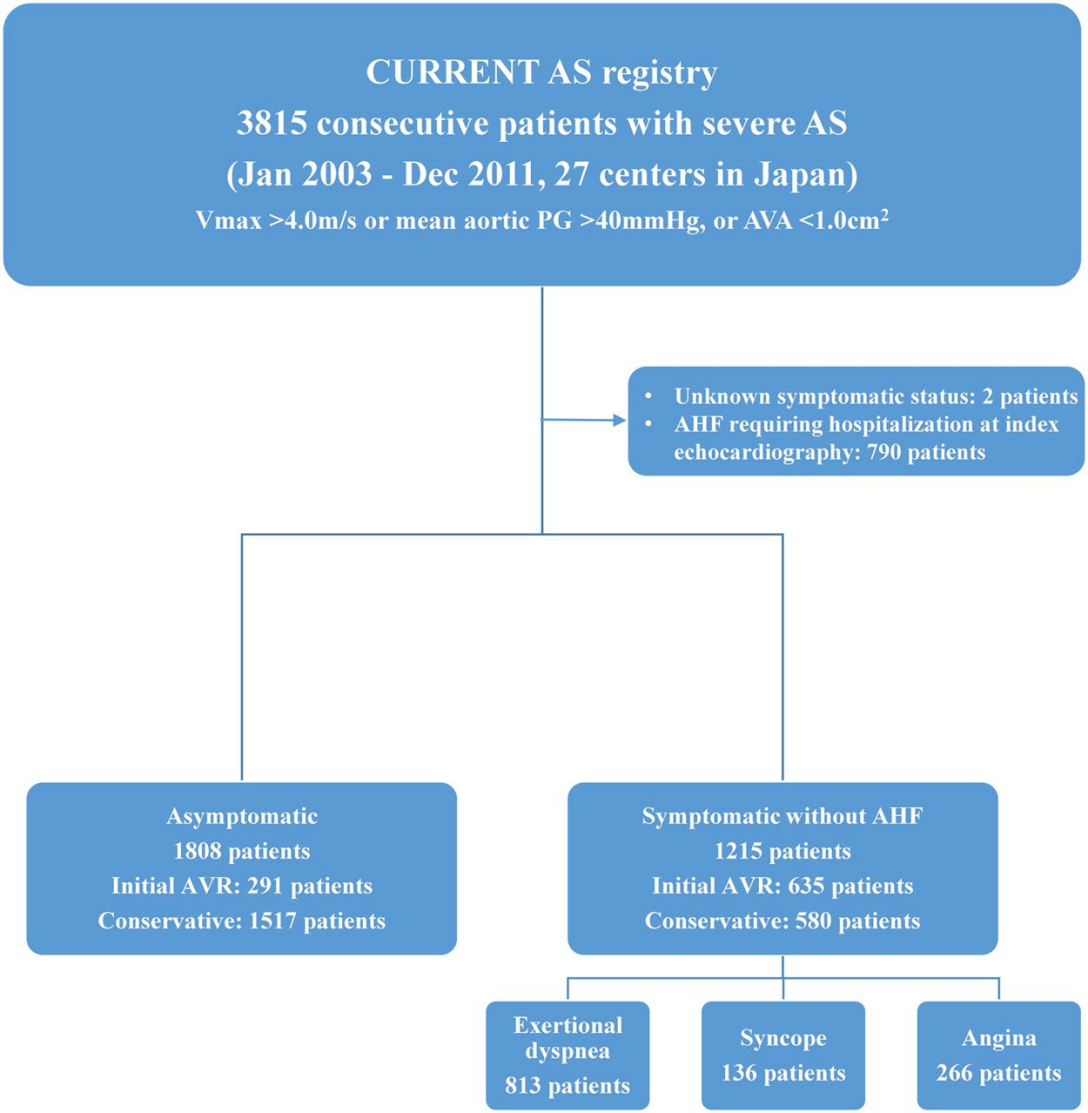
Study patient flow. AS = aortic stenosis; AHF = acute heart failure; AVA = aortic valve area; AVR = aortic valve replacement; PG = pressure gradient; Vmax = peak aortic jet velocity.

The symptoms related to AS at enrollment were classified into angina, syncope, and heart failure (HF) including AHF requiring hospitalization and chronic exertional dyspnea. Patients who had multiple types of symptoms were classified in one symptom category in the following priority order (AHF hospitalization > exertional dyspnea > syncope > angina). Among 3815 patients enrolled in this registry, 2005 (62.6%) patients had symptoms possibly related to AS (AHF hospitalization [N = 790, 20.7%]; exertional dyspnea [N = 813, 21.3%]; syncope [N = 136, 3.6%]; angina [N = 266, 7.0%]), while 1808 (47.4%) patients were asymptomatic, excluding 2 patients in whom the symptomatic status was not available. After the exclusion of 790 hospitalized patients due to AHF at baseline who were deemed to have an extremely high mortality risk, we compared the clinical outcomes between 1215 symptomatic patients without AHF hospitalization and 1808 asymptomatic patients. There were 926 patients (635 symptomatic patients [52.3%] and 291 asymptomatic [16.1%]), who were managed with the initial AVR strategy (Fig. 1). Follow-up was commenced on the day of index echocardiography, unless specified otherwise.

### Definitions of outcome measures

The primary outcome measure in the present analysis was a composite of aortic valve-related death or HF hospitalization. Other outcome measures included all-cause death, cardiovascular death, aortic valve-related death, aortic valve procedure death, sudden death, non-cardiovascular death and HF hospitalization, which were defined in line with the Valve Academic Research Consortium (VARC) -2 criteria. The causes of death were also classified according to the VARC-2 definitions^21,22^. The outcome measures and causes of death were adjudicated by a clinical event committee. HF hospitalization was defined as hospitalization due to worsening HF requiring intravenous drug therapy. Sudden death was defined as unexplained death in previously stable patients. Other definitions of clinical events have been described previously^13^.

### Statistical analysis

Categorical variables are presented as numbers and percentages, and were compared using the chi-squared test or Fisher’s exact test. Continuous variables were expressed as the mean ± SD or median with the interquartile range (IQR). Based on their distributions, continuous variables were compared using the Student’s t-test or Wilcoxon’s rank sum test. Inter-group comparisons according to various symptoms were performed using one-way ANOVA followed by the Tukey post hoc test or the Kruskal-Wallis test followed by the Steel-Dwass post hoc test for continuous variables and Bonferroni post hoc test for categorical variables. The cumulative incidences of events were estimated by the Kaplan-Meier method and the differences were assessed with the log-rank test. The risks of the symptomatic group relative to the asymptomatic group for the clinical outcome measures were assessed by the multivariable Cox proportional hazard models incorporating the initial AVR strategy and 18 clinically relevant covariates listed in Table 1 (age, sex, body mass index, hypertension, current smoking, diabetes on insulin, coronary artery disease, prior myocardial infarction, prior symptomatic stroke, aorta/peripheral artery disease, serum creatinine, hemodialysis, anemia, liver cirrhosis, malignancy currently under treatment, chronic lung disease, any valvular disease, and AS severity) as the risk-adjusting variables. The results were expressed as hazard ratios (HR) and their 95% confidence intervals (CI). The center was incorporated as the stratification variable. Consistent with our previous findings, continuous variables other than age were dichotomized by median values or clinically meaningful reference values. We used the same Kaplan-Meier method and Cox proportional hazard model to assess the risks for the clinical outcome measures according to the types of symptoms using the patients with angina as the reference. All statistical analyses were performed with the statistical software R 3.1.1 (The R Foundation for Statistical Computing, Vienna, Austria) or SAS 9.4 (SAS Institute Inc., Cary, North Carolina). All reported P values were 2-tailed, and P values < 0.05 were considered significant.

**Table 1:** Baseline characteristics and echocardiographic parameters.

	Symptomatic patients (N = 1215)	Asymptomatic patients (N = 1808)	P value
**Clinical characteristics**			
Age, years*	76.4 ± 9.8	76.8 ± 9.6	0.27
Age ≥ 80 years	484 (40%)	749 (41%)	0.39
Male*	469 (39%)	730 (40%)	0.34
BMI, kg/m²	22.0 ± 3.8	22.0 ± 3.8	0.71
BMI < 22 kg/m²*	698 (57%)	1057 (59%)	0.60
BSA, m²	1.47 ± 0.19	1.47 ± 0.18	0.96
Hypertension*	853 (70%)	1248 (69%)	0.49
Current smoking*	66 (5.4%)	95 (5.3%)	0.87
History of smoking	274 (23%)	402 (22%)	0.86
Dyslipidemia	464 (38%)	648 (36%)	0.19
On statin therapy	345 (28%)	464 (26%)	0.10
Diabetes mellitus	269 (22%)	434 (24%)	0.24
On insulin therapy*	63 (5.2%)	91 (5.0%)	0.87
Coronary artery disease*	406 (33%)	488 (27%)	<0.001
Prior myocardial infarction*	83 (6.8%)	151 (8.4%)	0.13
Prior PCI	128 (11%)	286 (16%)	<0.001
Prior CABG	70 (5.8%)	98 (5.4%)	0.69
Prior open heart surgery	101 (8.3%)	165 (9.1%)	0.47
Prior symptomatic stroke*	140 (12%)	253 (14%)	0.05
Atrial fibrillation or flutter	271 (22%)	338 (19%)	0.02
Aortic/peripheral vascular disease*	167 (14%)	299 (17%)	0.04
Serum creatinine, mg/dl*	0.87 (0.69–1.22)	0.84 (0.69–1.15)	0.35
Creatinine level > 2 mg/dl	184 (15%)	249 (14%)	0.29
Hemodialysis*	138 (11%)	207 (11%)	0.95
Anemia*	676 (56%)	862 (48%)	<0.001
Liver cirrhosis (Child-Pugh B or C)*	18 (1.5%)	11 (0.6%)	0.02
Malignancy	146 (12%)	276 (15%)	0.01
Malignancy currently under treatment*	33 (2.7%)	94 (5.2%)	0.001
Chest wall irradiation	10 (0.8%)	12 (0.7%)	0.67
Immunosuppressive therapy	34 (2.8%)	60 (3.3%)	0.46
Chronic lung disease (moderate or severe)*	39 (3.2%)	43 (2.4%)	0.17
Logistic EuroSCORE, %	8.6 (5.5–14.4)	8.4 (5.1–13.9)	0.08
EuroSCORE II,%	2.7 (1.5–4.1)	2.4 (1.4–3.6)	<0.001
STS score (PROM), %	3.4 (2.1–5.3)	3.3 (2.0–5.2)	0.23
Etiology of aortic stenosis			0.33
Degenerative	1058 (87%)	1584 (88%)	
Congenital (unicuspid, bicuspid, or quadricuspid)	95 (7.8%)	136 (7.5%)	
Rheumatic	54 (4.4%)	66 (3.7%)	
Infective endocarditis	0 (0%)	4 (0.2%)	
Other	8 (0.7%)	18 (1.0%)	
**Types of symptoms**			
Exertional dyspnea	813		
Syncope	163		
Angina	405		
Initial treatment strategy			<0.001
Initial AVR*	635 (52%)	291 (16%)	
Conservative	580 (48%)	1517 (84%)	
**Echocardiographic variables**			
Vmax, m/s	4.4 ± 0.9	3.9 ± 0.8	<0.001
Vmax ≥ 5 m/s	316 (26.0)	207 (11.5)	<0.001
Peak aortic PG, mmHg	81 ± 32	65 ± 28	<0.001
Mean aortic PG, mmHg	47 ± 21	36 ± 17	<0.001
Mean aortic PG ≥ 60 mmHg	253 (26%)	140 (9.4%)	<0.001
AVA (equation of continuity), cm²	0.68 ± 0.18	0.77 ± 0.17	<0.001
AVA index, cm²/m²	0.47 ± 0.13	0.53 ± 0.12	<0.001
AVA ≤ 0.6 cm^2^	416 (37%)	335 (20%)	<0.001
Eligibility for severe AS			
Vmax > 4 m/s*	851 (70%)	861 (48%)	<0.001
Mean aortic pressure gradient > 40 mmHg	573 (59%)	537 (36%)	<0.001
Vmax > 4 m/s or mean aortic PG > 40 mmHg	856 (71%)	869 (48%)	<0.001
AVA < 1.0 cm^2^ alone with LVEF < 50%	83 (6.8%)	111 (6.1%)	0.45
AVA < 1.0 cm^2^ alone with LVEF ≥ 50%	276 (23%)	828 (46%)	<0.001
LV end-diastolic diameter, mm	46.4 ± 7.1	44.8 ± 6.0	<0.001
LV end-systolic diameter, mm	30.3 ± 8.0	28.4 ± 6.2	<0.001
LVEF, %	63.4 ± 13.3	65.9 ± 10.9	<0.001
LVEF < 40%	83 (6.8%)	57 (3.2%)	<0.001
LVEF < 50%	172 (14%)	142 (7%)	<0.001
IVST in diastole	11.6 ± 2.3	11.1 ± 2.2	<0.001
PWT in diastole	11.2 ± 2.0	10.7 ± 2.0	<0.001
Any combined valvular disease (moderate or severe)*	522 (43%)	560 (31%)	<0.001
Moderate or severe AR	281 (23%)	293 (16%)	<0.001
Moderate or severe MS	54 (4.4%)	49 (2.7%)	0.01
Moderate or severe MR	250 (21%)	213 (12%)	<0.001
Moderate or severe TR	196 (16%)	216 (12%)	<0.001
TR pressure gradient ≥ 40 mm Hg	204 (17%)	173 (9.6%)	<0.001

Categorical variables were presented as number (percentage). Continuous variables were presented as mean ± SD, or median (interquartile range).

Anemia was defined as hemoglobin <12.0 g/dl in women and <13.0 g/dl in men.

Coronary artery disease included prior myocardial infarction, prior PCI, prior CABG, or documented coronary artery disease at baseline.

*Indicated the covariates incorporated in the multivariable Cox’s proportional hazard models as the risk-adjusting variables.

AR = aortic regurgitation; AS = aortic stenosis; AVA = aortic valve area; AVR = aortic valve replacement; BMI = body mass index; BSA = body surface area; CABG = coronary artery bypass grafting; IVST = interventricular septum thickness; LV = left ventricular; LVEF = left ventricular ejection fraction; MR = mitral regurgitation; MS = mitral stenosis; PCI = percutaneous coronary intervention; PG = pressure gradient; PROM = predicted risk of mortality; PWT = posterior wall thickness; STS = Society of Thoracic Surgeons; TR = tricuspid regurgitation; Vmax = peak aortic jet velocity.

## Results

### Baseline clinical characteristics

The baseline clinical characteristics were not so much different between symptomatic and asymptomatic patients, except for the significantly higher prevalence of coronary artery disease (CAD), atrial fibrillation/flutter, anemia, and liver cirrhosis, and the lower prevalence of prior percutaneous coronary intervention (PCI), aortic/peripheral disease and malignancy in symptomatic patients than in asymptomatic patients. Society of Thoracic Surgeons (STS) score and Logistic EuroSCORE were not different between the 2 groups, although EuroSCORE II was slightly but significantly higher in symptomatic patients than in asymptomatic patients (Table 1).

According to the types of symptoms, patients with angina had a higher prevalence of dyslipidemia, diabetes mellitus, CAD and prior PCI, as well as larger body surface area, while they had a lower prevalence of atrial fibrillation and chronic lung disease, as well as lower serum creatinine level and surgical risk scores than patients with exertional dyspnea. The baseline characteristics were generally similar between patients with angina and patients with syncope, except for the higher prevalence of diabetes mellitus, and CAD in the former (Supplementary Table 1).

### Baseline echocardiographic characteristics

Echocardiographic severity of AS, as evaluated by Vmax, mean aortic PG, and AVA, was greater in symptomatic patients than in asymptomatic patients. Symptomatic patients had lower left ventricular ejection fraction (LVEF), larger left ventricular dimensions, and greater left ventricular wall thickness than asymptomatic patients. The prevalence of combined valvular disease and estimated pressure gradient across the tricuspid valve were significantly higher in symptomatic patients than in asymptomatic patients (Table 1).

Patients with exertional dyspnea had smaller AVA, lower LVEF, larger left ventricular dimensions, higher estimated pressure gradient across the tricuspid valve, and higher prevalence of combined valvular disease than patients with angina. The echocardiographic parameters were generally similar between patients with angina and patients with syncope. (Supplementary Table 1).

### Clinical outcomes: Symptomatic patients versus asymptomatic patients

**Figure 2:**
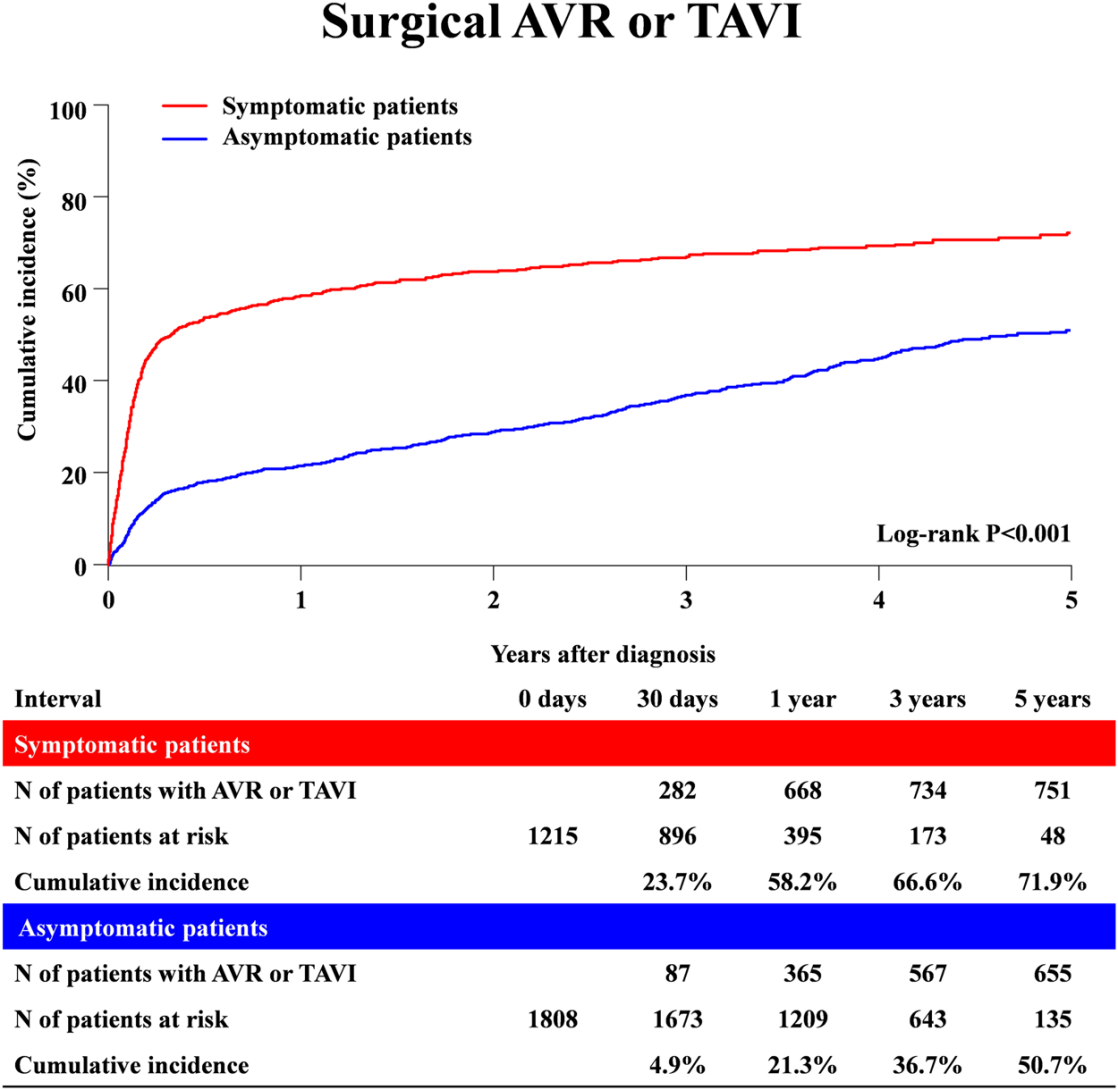
Cumulative incidence of surgical AVR or TAVI: Symptomatic versus Asymptomatic patients. AVR = aortic valve replacement; TAVI = transcatheter aortic valve implantation.

The reasons for non-referral to AVR in 580 symptomatic severe AS patients mainly included high risk for AVR in 240 patients (41.4%), and patient’s refusal in 163 patients (28.1%). On the other hand, among 291 patients referred for AVR despite absence of symptoms related to AS, 184 (63%) patients had 1 or more formal indications for AVR^13^. The median follow-up duration was 3.2 (IQR: 1.9–4.4) years with 95% follow-up at 2-year. During follow-up, 1399 patients (46.3%) underwent surgical AVR and 35 (1.2%) underwent transcatheter aortic valve implantation (TAVI). AVR or TAVI was more frequently performed in the symptomatic patients (755 of 1215 patients [62.1%], and cumulative 5-year incidence of 71.9% with a median interval of 42 [IQR: 19–100] days) than in the asymptomatic patients (679 of 1808 patients [37.6%], and cumulative 5-year incidence of 50.7% with a median interval of 269 [IQR: 51–897] days) (Fig. 2 and Table 2).

**Table 2:** Clinical outcomes: Symptomatic versus Asymptomatic patients.

	Symptomatic patients Number of patients with event (Cumulative 5-Year Incidence) N = 1215	Asymptomatic patients Number of patients with event (Cumulative 5-Year Incidence) N = 1808	Unadjusted HR (95% CI)	P Value	Adjusted HR (95% CI)	P Value
Composite of aortic valve-related death or hospitalization due to HF	315 (32.3%)	391(27.6%)	1.29 (1.11–1.49)	<0.001	1.64 (1.41–1.96)	<0.001
All-cause death	425 (39.4%)	582 (37.5%)	1.15 (0.98–1.26)	0.09	1.34 (1.16–1.54)	<0.001
Cardiovascular death	279 (27.9%)	348 (25.0%)	1.23 (1.05–1.44)	0.01	1.42 (1.19–1.69)	<0.001
Aortic valve-related death	156 (16.1%)	210 (16.6%)	1.14 (0.92–1.40)	0.23	1.38 (1.10–1.74)	0.006
Aortic valve-procedure death	31 (2.7%)	24 (2.0%)	1.98 (1.16–3.37)	0.01	N/A	—
Sudden death	53 (5.2%)	90 (7.0%)	0.90 (0.64–1.26)	0.54	1.11 (0.76–1.62)	0.59
Non-cardiovascular death	146 (16.0%)	234 (16.7%)	0.95 (0.77–1.17)	0.61	1.19 (0.94–1.50)	0.16
HF hospitalization	250 (28.2%)	294 (21.5%)	1.37 (1.15–1.62)	<0.001	1.85 (1.54–2.24)	<0.001
Surgical AVR or TAVI	755 (71.9%)	679 (50.7%)	2.63 (2.37–2.92)	<0.001	N/A	—

The number of patients with event was counted through the entire follow-up period, while the cumulative 5-year incidence was truncated at 5-year.

Any death during hospitalization for AVR or TAVI was regarded as aortic procedure-related death. Aortic valve-related death included aortic procedure-related death, sudden death, and death due to HF. HF hospitalization was defined as hospitalization due to worsening HF requiring intravenous drug therapy.

Risk-adjusting variables: Initial AVR strategy and 18 clinically relevant risk-adjusting variables: age, sex, body mass index, hypertension, current smoking, diabetes on insulin, coronary artery disease, prior myocardial infarction, prior symptomatic stroke, aorta/peripheral artery disease, serum creatinine, hemodialysis, anemia, liver cirrhosis, malignancy currently under treatment, chronic lung disease, any valvular disease, and AS severity.

AS = aortic stenosis; AVR = aortic valve replacement; CI = confidence interval; HF = heart failure; HR = hazard ratio; N/A = not assessed; TAVI = transcatheter aortic valve implantation.

**Figure 3:**
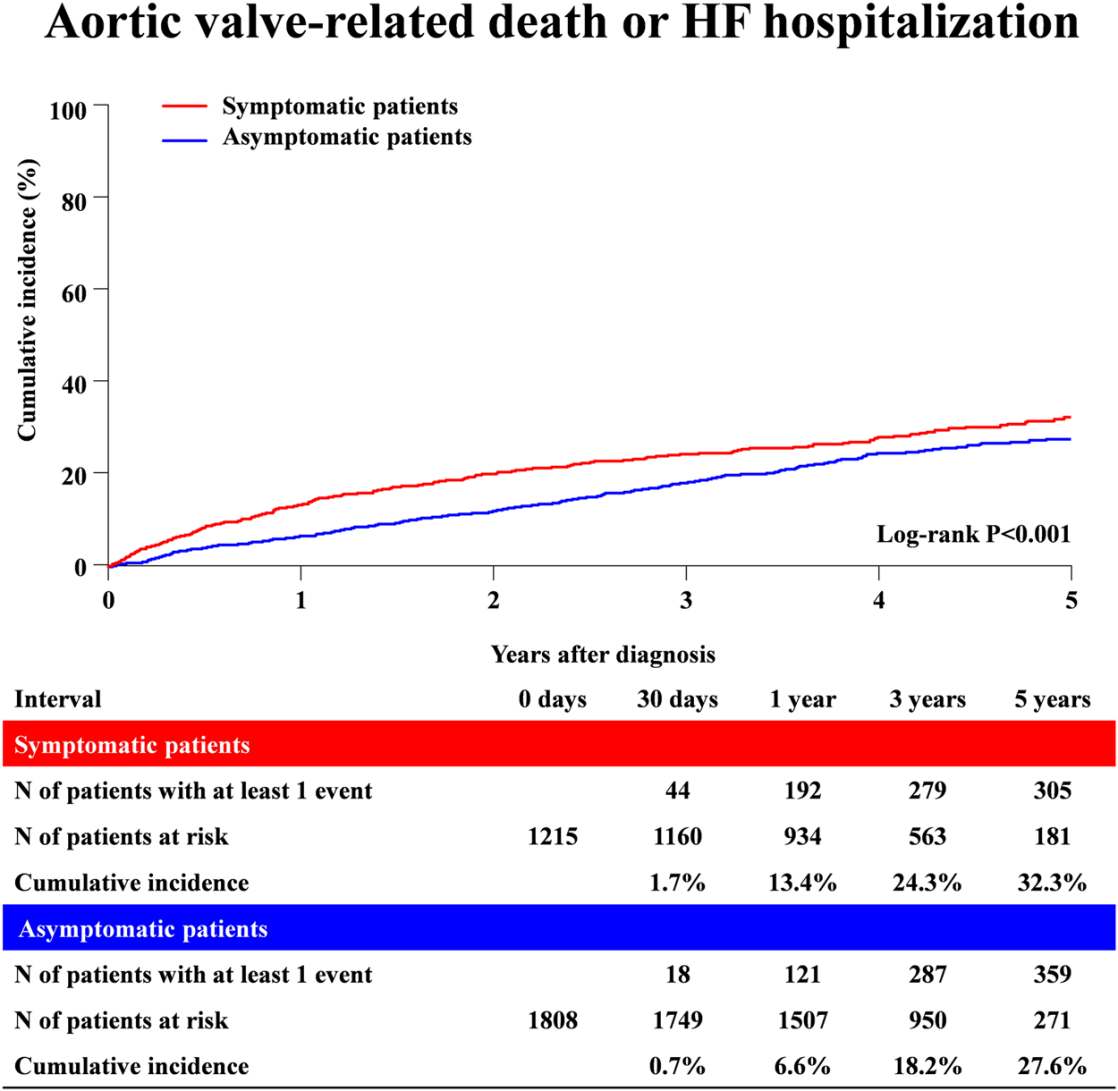
Cumulative incidence of the primary outcome measure (aortic valve-related death or HF hospitalization): Symptomatic versus Asymptomatic patients. HF = heart failure.

A total of 1007 (33%) out of 3023 patients died during follow-up, with HF (194 patients) and sudden death (143 patients) being the dominant causes (Supplementary Table 2). The cumulative incidence of the primary outcome measure (a composite of aortic valve-related death or HF hospitalization) was modestly but significantly higher in symptomatic patients than in asymptomatic patients (32.3% versus 27.6%, P < 0.001) (Fig. 3 and Table 2). However, the cumulative incidences of all-cause death, aortic valve-related death, and sudden death in asymptomatic patients remained high and not different from those in symptomatic patients (Table 2).

In the adjusted analysis, the excess risk of symptomatic patients relative to the asymptomatic patients for the primary outcome measure remained significant (HR 1.64, 95% CI; 1.41–1.96, P < 0.001) (Table 2). Also, the excess adjusted risks of symptomatic patients relative to asymptomatic patients were significant for all-cause death, cardiovascular death, aortic valve-related death and HF hospitalization (Table 2).

### Clinical outcomes for each symptoms

AVR or TAVI was less frequently performed in patients with exertional dyspnea than in patients with syncope and angina (Supplementary Table 3). The crude cumulative 5-year incidence of the primary outcome measure in patients with exertional dyspnea, syncope and angina was 37.6%, 25.8% and 19.4%, respectively (Supplementary Figure). In the adjusted analysis, patients with exertional dyspnea had significantly higher risk for the primary outcome measure (HR 1.75, 95% CI; 1.23–2.47, P = 0.002) than patients with angina (Supplementary Table 3). The risk for the primary outcome measure was not different between patients with syncope and patients with angina (HR 1.44, 95% CI; 0.89–2.35, P = 0.14) (Supplementary Table 3).

## Discussion

The main findings of the present study were the followings; (1) Symptomatic patients with severe AS had greater echocardiographic severity of AS, and more depressed left ventricular function than asymptomatic patients with severe AS without much difference in other baseline clinical characteristics and surgical risk scores; (2) Nearly 40% of the symptomatic patients did not receive AVR despite the presence of symptoms, while nearly 40% of the asymptomatic patients underwent AVR during 5-year follow-up; (3) Even after excluding those patients with AHF at baseline, the excess risk of symptomatic patients relative to asymptomatic patients for the aortic valve related events (a composite of aortic valve-related death or HF hospitalization) was significant with or without multivariable adjustment for AVR and other risk-adjusting variables; (4) Crude cumulative 5-year incidences of aortic valve-related death, and sudden death in asymptomatic patients remained high and similar to those in symptomatic patients.

Patients with severe AS is becoming markedly older and having multiple comorbidities, as the etiology of AS has changed from rheumatic disease to calcified degeneration. In the present study, the baseline clinical characteristics and surgical risk scores were almost comparable between asymptomatic and symptomatic patients with severe AS, although there was a slight but significant excess of CAD, atrial fibrillation and anemia in symptomatic patients. On the other hands, symptomatic patients had greater echocardiographic severity of aortic stenosis, and more depressed left ventricular function than asymptomatic patients, although Park *et al*. reported that there was no difference in Vmax, mean aortic PG and AVA between symptomatic and asymptomatic patients with severe AS^23^. The difference between the 2 studies might be related to the small number of patients in their study as well as the exclusion of patients with AHF in our study. Furthermore, patients presenting with exertional dyspnea had more depressed left ventricular function as indicated by left ventricular dilation and reduced ejection fraction than patients presenting with syncope or angina.

Even after excluding those patients with AHF at baseline, the excess risk of symptomatic patients relative to asymptomatic patients for the aortic valve related events was significant with or without multivariable adjustment for AVR and other risk-adjusting variables. One of the reasons for the poor prognosis of symptomatic patients might be related to greater AS severity and more depressed left ventricular function in symptomatic patients. Indeed, we have recently reported that the long-term outcomes of symptomatic patients with severe AS were worse than those of asymptomatic patients when managed with initial AVR strategy^16^. Therefore, the watchful waiting strategy might be associated with poorer prognosis than early AVR strategy, even if elective AVR could be performed after watchful waiting. Another reason would be related to the relatively low rate of AVR in symptomatic patients, which is consistent with several previous reports^8,24,25^. Therefore, we should further promote implementation of AVR for symptomatic patients to improve their long-term outcomes, desirably before incurring irreversible myocardial fibrosis. TAVI might have already changed the landscape in the treatment of symptomatic AS patients with high or prohibitive surgical risk^9,26^.

In the present study, asymptomatic patients with severe AS had lower risk for AS-related events than symptomatic patients with AVR performed in 38% of patients during follow-up. However, crude cumulative 5-year incidences of aortic valve-related death, and sudden death in asymptomatic patients remained high and similar to those in symptomatic patients. Therefore, the long-term clinical outcomes of asymptomatic patients with severe AS look far from acceptable. Regarding the symptoms related to AS, some severe AS patients with a decrease in daily living activity may not have recognizable symptoms. Thus, physicians may underestimate the presence of symptoms in severe AS patients. Our previous report suggested that the initial AVR strategy might improve the clinical outcomes in asymptomatic patients with severe AS^13^. The risk of serious AS-related events may be low in truly asymptomatic patients with a normal exercise stress test^27^. We could not exclude the possibility of ascertainment bias for symptoms related to AS at baseline, although we thoroughly reviewed all patient charts and referred to the hospital database to evaluate symptomatic status. It could be possible that a symptomatic patient might have been included in the asymptomatic group, because exercise test was rarely performed to ensure that patients were truly asymptomatic. There might be patients who just could not physically exert themselves due to symptomatic AS but was labeled as “asymptomatic AS”. These patients might have contributed to the higher incidence of bad clinical outcomes in the asymptomatic AS group. However, the availability of objective echocardiographic parameters may be useful for identifying subjects at a higher risk of adverse events among asymptomatic patients with severe AS^19,28^.

There are several limitations in the present study. First, we were unable to exclude the possibility of ascertainment bias for symptoms related to AS at baseline and decisions for the initial AVR strategy were not uniform because of the retrospective nature of this study. Second, care is needed regarding the interpretation of adjusted results. We did not include left ventricular function, pulmonary hypertension, or atrial fibrillation as the risk-adjusting variables in the multivariable Cox model, because these factors are related to the evolution of symptoms. Third, the possible imprecision of assessing AVA by echocardiography with the overestimation of AS severity in some patients might represent a potential concern. Finally, most of this study period was the era before TAVI introduction in Japan. Therefore, TAVI could not be performed in high-risk patients.

## Conclusions

The excess risk of symptomatic relative to asymptomatic patients with severe AS for the aortic valve-related event was significant. However, the prevalence of AVR in symptomatic patients was not optimal, and the long-term aortic valve-related event rate in asymptomatic patients remained unacceptably high.

## Electronic supplementary material

Supplementary materials
