# Supplementary material for: Asymptomatic versus Symptomatic Patients with Severe Aortic Stenosis
Source: Sci Rep. 2018 Jul 4;8:10080. doi: 10.1038/s41598-018-28162-x (PMC6031663; doi:10.1038/s41598-018-28162-x)

**Supplementary materials**

**Title: Asymptomatic versus Symptomatic Patients with Severe Aortic Stenosis**

**Authors**: Norio Kanamori, MD^1^; Tomohiko Taniguchi, MD^2^; Takeshi Morimoto, MD, MPH^3^; Hiroki Shiomi, MD^2^; Kenji Ando, MD^4^; Koichiro Murata, MD^5^; Takeshi Kitai, MD^6^; Yuichi Kawase, MD^7^; Chisato Izumi, MD^8^; Makoto Miyake, MD^8^; Hirokazu Mitsuoka, MD^9^; Masashi Kato, MD^10^; Yutaka Hirano, MD^11^; Shintaro Matsuda, MD^2^; Kazuya Nagao, MD^12^; Tsukasa Inada, MD^12^; Hiroshi Mabuchi, MD^13^; Yasuyo Takeuchi, MD^14^; Keiichiro Yamane, MD^15^; Mamoru Toyofuku, MD^16^; Mitsuru Ishii, MD^17^; Eri Minamino-Muta, MD^2^; Takao Kato, MD^2^; Moriaki Inoko, MD^18^; Tomoyuki Ikeda, MD^19^; Akihiro Komasa, MD^20^; Katsuhisa Ishii, MD^20^; Kozo Hotta, MD^21^; Nobuya Higashitani, MD^22^; Yoshihiro Kato, MD^23^; Yasutaka Inuzuka, MD^24^; Chiyo Maeda, MD^25^; Toshikazu Jinnai, MD^22^; Yuko Morikami, MD^26^; Naritatsu Saito^2^, MD; Kenji Minatoya, MD^27^; Takeshi Aoyama, MD^1^; Takeshi Kimura, MD^2*^, on behalf of the CURRENT AS registry Investigators

**Supplementary material content**

**Supplementary Tables Page 3-13**

**Supplementary Figures Page 14-15**

Supplementary Table 1. Baseline characteristics according to symptom

|  | **Exertional dyspnea**  **(N=813)** | **Syncope**  **(N=136)** | **Angina**  **(N=266)** | **P value ‖** |
| --- | --- | --- | --- | --- |
| **Clinical characteristics** |  |  |  |  |
| Age, years * | 76.7±10.0 | 77.1±10.3 | 75.4±8.8 | 0.12 |
| Age ≥80 years | 338 (42%) † | 61 (45%) † | 85 (32%) | 0.009 |
| Male * | 303 (37%) | 52 (38%) | 114 (43%) | 0.27 |
| BMI, kg/m² | 21.9±3.8 | 21.9±3.7 | 22.4±3.6 | 0.22 |
| BMI <22 kg/m² * | 475 (58%) | 86 (63%) | 137 (52%) | 0.50 |
| BSA, m² | 1.46±0.19 † | 1.47±0.18 | 1.50±0.17 | 0.02 |
| Hypertension * | 569 (7%) | 96 (71%) | 188 (71%) | 0.97 |
| Current smoking * | 36 (4.4%) | 10 (7.4%) | 20 (7.5%) | 0.09 |
| History of smoking | 172 (21%) | 35 (26%) | 67 (25%) | 0.25 |
| Dyslipidemia | 288 (35%) ‡ | 49 (36%) | 127 (48%) | 0.001 |
| On statin therapy | 212 (26%) ‡ | 37 (27%) | 96 (36%) | 0.007 |
| Diabetes mellitus | 166 (20%) ‡ | 22 (16%) ‡ | 81 (31%) | 0.001 |
| On insulin therapy * | 35 (4.3%) | 10 (7.4%) | 18 (6.8%) | 0.14 |
| Coronary artery disease * | 238 (29.3%) § | 45 (33.1%) † | 123 (46.2%) | <0.001 |
| Prior myocardial infarction * | 60 (7.4%) | 4 (2.9%) | 19 (7.1%) | 0.15 |
| Prior PCI | 74 (9.1%) † | 13 (9.6%) | 41 (15%) | 0.02 |
| Prior CABG | 49 (6.0%) | 5 (3.7%) | 16 (6.0%) | 0.60 |
| Prior open heart surgery | 76 (9.3%) | 7 (5.1%) | 18 (6.8%) | 0.17 |
| Prior symptomatic stroke * | 89 (11%) | 23 (17%) | 28 (11%) | 0.12 |
| Atrial fibrillation or flutter | 212 (26%) § | 24 (18%) | 35 (13%) | <0.001 |
| Aortic/peripheral vascular disease * | 107 (13%) | 17 (13%) | 43 (16%) | 0.43 |
| Serum creatinine, mg/dl * | 0.90 (0.70-1.28) † | 0.87 (0.69-1.39) | 0.80 (0.66-1.07) | 0.03 |
| Creatinine level >2 mg/dl | 121 (15%) | 23 (17%) | 40 (15%) | 0.80 |
| Hemodialysis * | 83 (10%) | 18 (13%) | 37 (14%) | 0.19 |
| Anemia * | 451 (56%) | 84 (62%) | 141 (53%) | 0.25 |
| Liver cirrhosis (Child-Pugh B or C) * | 12 (1.5%) | 2 (1.5%) | 4 (1.5%) | 1.0 |
| Malignancy | 89 (11%) | 22 (16%) | 35 (13%) | 0.17 |
| Malignancy currently under treatment * | 22 (2.7%) | 2 (1.5%) | 9 (3.4%) | 0.54 |
| Chest wall irradiation | 8 (1.0%) | 1 (0.7%) | 1 (0.4%) | 0.79 |
| Immunosuppressive therapy | 23 (2.8%) | 5 (3.7%) | 6 (2.3%) | 0.69 |
| Chronic lung disease (moderate or severe) * | 35 (4.3%) ‡ | 2 (1.5%) | 2 (0.8%) | 0.005 |
| Logistic EuroSCORE, % | 9.5 (5.5-15.6) § | 9.0 (5.4-12.8) | 7.5 (5.1-11.9) | <0.001 |
| EuroSCORE II, % | 3.0 (1.8-4.51) § | 2.5 (1.4-3.4) | 1.9 (1.3-3.1) | <0.001 |
| STS score (PROM), % | 3.5 (2.1-5.7) † | 3.2 (2.2-5.2) | 2.9 (1.9-4.8) | 0.01 |
| Etiology of aortic stenosis |  |  |  | 0.64 |
| Degenerative | 704 (87%) | 122 (90%) | 232 (87%) |  |
| Congenital (unicuspid, bicuspid, or quadricuspid) | 57 (7.0%) | 10 (7.4%) | 28 (10.5%) |  |
| Rheumatic | 46 (5.7%) | 3 (2.2%) | 5 (1.9%) |  |
| Infective endocarditis | 0 (0%) | 0 (0%) | 0 (0%) |  |
| Other | 6 (0.7%) | 1 (0.7%) | 1 (0.4%) |  |
| Initial treatment strategy |  |  |  | <0.001 |
| Initial AVR * | 389 (48%) § | 79 (58%) | 167 (63%) |  |
| Conservative | 424 (52%) § | 57 (42%) | 99 (37%) |  |
| **Echocardiographic variables** |  |  |  |  |
| Vmax, m/s | 4.4±1.0 | 4.6±1.0 | 4.4±0.8 | 0.09 |
| Vmax ≥5 m/s | 217 (27%) | 44 (32%) † | 55 (21%) | 0.03 |
| Peak aortic PG, mmHg | 81±33 | 87±36 | 79±28 | 0.05 |
| Mean aortic PG, mmHg | 47±21 | 50±22 | 46±18 | 0.39 |
| Mean aortic PG ≥60 mmHg | 178 (27%) | 26 (26%) | 49 (23%) | 0.54 |
| AVA (equation of continuity), cm² | 0.68±0.18 † | 0.67±0.17 | 0.71±0.18 | 0.02 |
| AVA index, cm²/m² | 0.47±0.13 | 0.47±0.12 | 0.48±0.12 | 0.27 |
| AVA ≤0.6 cm^2^ | 295 (39%) ‡ | 51 (40%) | 70 (27%) | 0.005 |
| Eligibility for severe AS |  |  |  |  |
| Vmax >4m/s * | 563 (69%) | 102 (75%) | 186 (70%) | 0.40 |
| Mean aortic pressure gradient >40 mmHg | 382 (58%) | 63 (63%) | 128 (61%) | 0.61 |
| Vmax >4 m/s or mean aortic PG >40 mmHg | 567 (70%) | 102 (75%) | 187 (70%) | 0.47 |
| AVA <1.0 cm^2^ alone with LVEF <50% | 74 (9.1%) ‡ | 1 (0.7%) | 8 (3.0%) | <0.001 |
| AVA <1.0 cm^2^ alone with LVEF ≥50% | 172 (21%) | 33 (24%) | 71 (27%) | 0.15 |
| LV end-diastolic diameter, mm | 46.92±7.38 ‡ | 44.98±6.27 | 45.35±6.34 | <0.001 |
| LV end-systolic diameter, mm | 31.28±8.50 § | 28.76±6.18 | 28.21±6.70 | <0.001 |
| LVEF, % | 61.5±14.2 § | 66.0±9.7 | 68.1±10.3 | <0.001 |
| LVEF <40% | 78 (9.6%) § | 1 (0.7%) | 4 (1.5%) | <0.001 |
| LVEF <50% | 151 (18.6%) § | 6 (4.4%) | 15 (5.6%) | <0.001 |
| IVST in diastole | 11.57±2.31 | 11.79±2.54 | 11.65±2.26 | 0.57 |
| PWT in diastole | 11.23±2.03 | 11.11±1.99 | 11.26±1.84 | 0.75 |
| Any combined valvular disease  (moderate or severe) * | 402 (49%) † | 50 (37%) | 70 (26%) | <0.001 |
| Moderate or severe AR | 209 (26%) † | 26 (19%) | 46 (17%) | 0.009 |
| Moderate or severe MS | 47 (5.8%) † | 2 (1.5%) | 5 (1.9%) | 0.005 |
| Moderate or severe MR | 196 (24%) § | 26 (19%) | 28 (11%) | <0.001 |
| Moderate or severe TR | 163 (20%) § | 13 (9.6%) | 20 (7.5%) | <0.001 |
| TR pressure gradient ≥40mm Hg | 172 (21%) § | 13 (9.6%) | 19 (7.1%) | <0.001 |

Categorical variables were presented as number (percentage). Continuous variables were presented as mean ± SD, or median (interquartile range).

Anemia was defined as hemoglobin <12.0 g/dl in women and <13.0 g/dl in men.

Coronary artery disease included prior myocardial infarction, prior PCI, prior CABG, or documented coronary artery disease at baseline.

* Indicated the covariates incorporated in the multivariable Cox’s proportional hazard models as the risk-adjusting variables.

AR=aortic regurgitation; AS=aortic stenosis; AVA=aortic valve area; AVR=aortic valve replacement; BMI=body mass index; BSA=body surface area; CABG=coronary artery bypass grafting; IVST=interventricular septum thickness; LV=left ventricular; LVEF=left ventricular ejection fraction; MR=mitral regurgitation; MS=mitral stenosis; PCI=percutaneous coronary intervention; PG=pressure gradient; PROM=predicted risk of mortality; PWT=posterior wall thickness; STS=Society of Thoracic Surgeons; TR=tricuspid regurgitation; Vmax=peak aortic jet velocity.

Significant difference († P<0.05, ‡ P<0.01 and § P<0.001) in the post hoc comparisons with Angina group.

‖ Comparison between 3 groups.

Supplementary Table 2. Causes of death

|  | **Symptomatic patients**  **Number of patients**  **(Proportion)**  **N=1215** | **Asymptomatic patients**  **Number of patients**  **(Proportion)**  **N=1808** |
| --- | --- | --- |
| All-cause death | 425 | 582 |
| Cardiovascular death | 279 (66%) | 348 (60%) |
| Heart failure | 92 | 102 |
| Aortic valve-procedure death | 31 | 24 |
| Myocardial infarction | 10 | 9 |
| Sudden death | 53 | 90 |
| Infective endocarditis | 2 | 6 |
| Stroke | 26 | 30 |
| Renal failure | 15 | 19 |
| Aortic/peripheral vascular disease | 10 | 21 |
| Other cardiac cause | 1 | 3 |
| Unknown death | 39 | 44 |
| Non-cardiovascular death | 146 (34%) | 234 (40%) |
| Malignancy | 54 | 82 |
| Infection | 45 | 88 |
| Respiratory failure | 6 | 15 |
| Liver failure | 5 | 4 |
| Bleeding | 4 | 5 |
| Trauma | 2 | 4 |
| Others | 30 | 36 |

Supplementary Table 3. Clinical outcomes for each symptoms

|  | **Number of patients**  **with event**  **(Cumulative 5-Year Incidence [%])** | **Log-rank**  **P Value** | **Unadjusted HR (95% CI)** | **P Value** | **Adjusted HR (95% CI)** | **P Value** |
| --- | --- | --- | --- | --- | --- | --- |
| Composite of aortic valve-related death or hospitalization due to HF |  |  |  |  |  |  |
| Angina (N=266) | 46 (19.4%) | <0.001 | 1 (reference) |  | 1 (reference) |  |
| Syncope (N=136) | 28 (25.8%) |  | 1.31 (0.82-2.10) | 0.26 | 1.44 (0.89-2.35) | 0.14 |
| Exertional dyspnea (N=813) | 241 (37.6%) |  | 1.94 (1.42-2.66) | <0.001 | 1.75 (1.23-2.47) | 0.002 |
| All-cause death |  |  |  |  |  |  |
| Angina | 72 (30.0%) | 0.01 | 1 (reference) |  | 1 (reference) |  |
| Syncope | 49 (45.6%) |  | 1.43 (0.99-2.05) | 0.06 | 1.53 (1.03-2.26) | 0.03 |
| Exertional dyspnea | 304 (41.4%) |  | 1.47 (1.14-1.91) | 0.003 | 1.32 (0.99-1.75) | 0.06 |
| Cardiovascular death |  |  |  |  |  |  |
| Angina | 44 (19.2%) | 0.02 | 1 (reference) |  | 1 (reference) |  |
| Syncope | 34 (33.0%) |  | 1.62 (1.03-2.53) | 0.04 | 1.73 (1.07-2.80) | 0.03 |
| Exertional dyspnea | 201 (29.9%) |  | 1.59 (1.15-2.21) | 0.005 | 1.43 (0.99-2.06) | 0.06 |
| Aortic valve-related death |  |  |  |  |  |  |
| Angina | 25 (12.1%) | 0.11 | 1 (reference) |  | 1 (reference) |  |
| Syncope | 18 (17.0%) |  | 1.53 (0.83-2.80) | 0.17 | 1.79 (0.94-3.43) | 0.08 |
| Exertional dyspnea | 113 (17.4%) |  | 1.58 (1.02-2.43) | 0.04 | 1.48 (0.90-2.43) | 0.12 |
| Aortic valve-procedure death |  |  |  |  |  |  |
| Angina | 4 (1.2%) | 0.18 | 1 (reference) |  | N/A |  |
| Syncope | 6 (4.9%) |  | 3.16 (0.89-11.2) | 0.07 | N/A |  |
| Exertional dyspnea | 21 (2.9%) |  | 1.83 (0.63-5.34) | 0.27 | N/A |  |
| Sudden death |  |  |  |  |  |  |
| Angina | 11 (3.4%) | 0.85 | 1 (reference) |  | N/A |  |
| Syncope | 7 (9.3%) |  | 1.34 (0.52-3.46) | 0.54 | N/A |  |
| Exertional dyspnea | 35 (5.2%) |  | 1.09 (0.55-2.15) | 0.81 | N/A |  |
| Non-cardiovascular death |  |  |  |  |  |  |
| Angina | 28 (13.3%) | 0.46 | 1 (reference) |  | 1 (reference) |  |
| Syncope | 15 (18.8%) |  | 1.13 (0.60-2.12) | 0.70 | 1.13 (0.56-2.28) | 0.73 |
| Exertional dyspnea | 103 (16.5%) |  | 1.30 (0.85-1.96) | 0.23 | 1.13 (0.71-1.81) | 0.61 |
| HF hospitalization |  |  |  |  |  |  |
| Angina | 33 (16.4%) | <0.001 | 1 (reference) |  | 1 (reference) |  |
| Syncope | 17 (18.1%) |  | 1.12 (0.63-2.01) | 0.70 | 1.28 (0.69-2.35) | 0.43 |
| Exertional dyspnea | 200 (33.8%) |  | 2.27 (1.57-3.28) | <0.001 | 1.97 (1.31-3.00) | 0.001 |
| Surgical AVR or TAVI |  |  |  |  |  |  |
| Angina | 189 (77.3%) | 0.002 | 1 (reference) |  | N/A |  |
| Syncope | 87 (75.3%) |  | 0.97 (0.75-1.25) | 0.82 | N/A |  |
| Exertional dyspnea | 479 (69.5%) |  | 0.76 (0.64-0.90) | 0.001 | N/A |  |

The number of patients with event was counted through the entire follow-up period, while the cumulative 5-year incidence was truncated at 5-year.

Any death during hospitalization for AVR or TAVI was regarded as aortic procedure-related death. Aortic valve-related death included aortic procedure-related death, sudden death, and death due to HF. HF hospitalization was defined as hospitalization due to worsening HF requiring intravenous drug therapy.

Risk-adjusting variables: Initial AVR strategy and 18 clinically relevant risk-adjusting variables: age, sex, body mass index, hypertension, current smoking, diabetes on insulin, coronary artery disease, prior myocardial infarction, prior symptomatic stroke, aorta/peripheral artery disease, serum creatinine, hemodialysis, anemia, liver cirrhosis, malignancy currently under treatment, chronic lung disease, any valvular disease, and AS severity.

AS=aortic stenosis; AVR=aortic valve replacement; CI=confidence interval; HF=heart failure; HR=hazard ratio; N/A=not assessed; TAVI=transcatheter aortic valve implantation.

**Supplementary figure legend**

Supplementary Figure. Cumulative incidence of the primary outcome measure (aortic valve-related death or HF hospitalization) for each symptoms.

HF=heart failure.

Supplementary Figure.


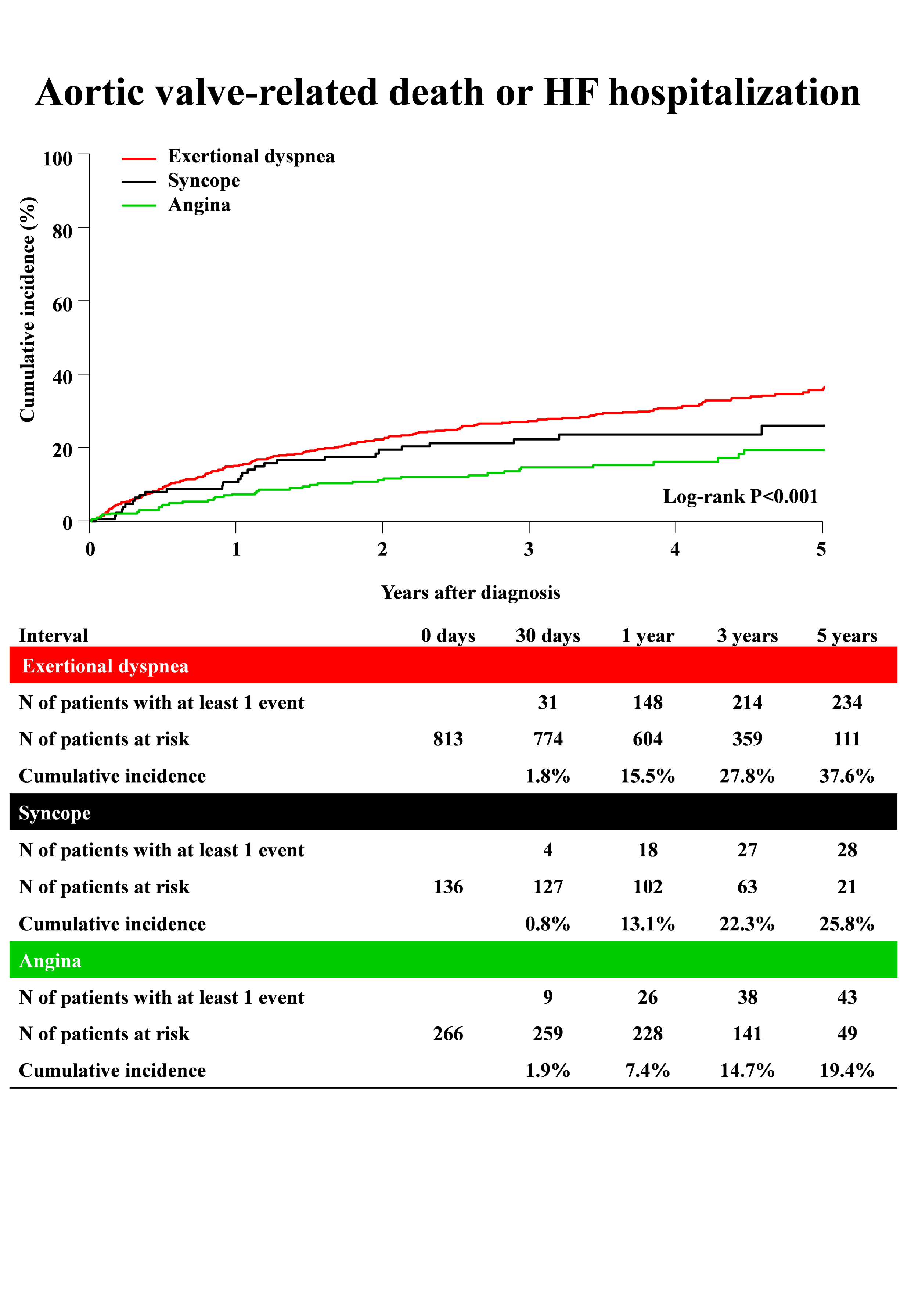

Supplement: Supplementary file 1 — Supplementary materials [file 41598_2018_28162_MOESM1_ESM.doc]
